# Coincident Resection at Both Ends of Random, γ–Induced Double-Strand Breaks Requires MRX (MRN), Sae2 (Ctp1), and Mre11-Nuclease

**DOI:** 10.1371/journal.pgen.1003420

**Published:** 2013-03-28

**Authors:** James W. Westmoreland, Michael A. Resnick

**Affiliations:** Chromosome Stability Section, Laboratory of Molecular Genetics, National Institute of Environmental Health Sciences, National Institutes of Health, Research Triangle Park, North Carolina, United States of America; National Cancer Institute, United States of America

## Abstract

Resection is an early step in homology-directed recombinational repair (HDRR) of DNA double-strand breaks (DSBs). Resection enables strand invasion as well as reannealing following DNA synthesis across a DSB to assure efficient HDRR. While resection of only one end could result in genome instability, it has not been feasible to address events at both ends of a DSB, or to distinguish 1- versus 2-end resections at random, radiation-induced “dirty” DSBs or even enzyme-induced “clean” DSBs. Previously, we quantitatively addressed resection and the role of Mre11/Rad50/Xrs2 complex (MRX) at random DSBs in circular chromosomes within budding yeast based on reduced pulsed-field gel electrophoretic mobility (“PFGE-shift”). Here, we extend PFGE analysis to a second dimension and demonstrate unique patterns associated with 0-, 1-, and 2-end resections at DSBs, providing opportunities to examine coincidence of resection. In G2-arrested WT, *Δrad51* and *Δrad52* cells deficient in late stages of HDRR, resection occurs at both ends of γ-DSBs. However, for radiation-induced and I-*Sce*I-induced DSBs, 1-end resections predominate in MRX (MRN) null mutants with or without Ku70. Surprisingly, Sae2 (Ctp1/CtIP) and Mre11 nuclease-deficient mutants have similar responses, although there is less impact on repair. Thus, we provide direct molecular characterization of coincident resection at random, radiation-induced DSBs and show that rapid and coincident initiation of resection at γ-DSBs requires MRX, Sae2 protein, and Mre11 nuclease. Structural features of MRX complex are consistent with coincident resection being due to an ability to interact with both DSB ends to directly coordinate resection. Interestingly, coincident resection at clean I-*Sce*I-induced breaks is much less dependent on Mre11 nuclease or Sae2, contrary to a strong dependence on MRX complex, suggesting different roles for these functions at “dirty” and clean DSB ends. These approaches apply to resection at other DSBs. Given evolutionary conservation, the observations are relevant to DNA repair in human cells.

## Author Summary

DNA double-strand breaks (DSBs) can cause genome instability and cancer. While repair can occur through recombination, coincident events at both ends—while assumed—have not been directly addressable at unique or random damage-induced DSBs. Here, we describe pulse-field gel electrophoresis approaches that for the first time distinguish resection at 0, 1, or both ends of DSBs. Resection, an early step in DSB end-processing, is efficiently initiated at both ends of random, radiation-induced DSBs in wild-type budding yeast and in cells deficient in late steps of recombinational repair. However, 0- and 1-end resections predominate in MRX-null, Sae2, and Mre11 nuclease mutants, suggesting new roles for the cancer-related proteins (Ctp1 and MRN in humans) in repair, namely, efficient and coincident resection at both ends of a DSB. We suggest that the structural features of the MRX complex are consistent with coincident resection being due to an ability to interact with both DSB ends to directly coordinate resection. Interestingly, we provide direct evidence that coincident resection at a clean I-*Sce*I-induced break is much less dependent on the Mre11 nuclease or Sae2, contrary to the strong dependence on the MRX complex. These results suggest a differential role for these functions at “dirty” and clean DSB ends.

## Introduction

Repair of DSBs is intrinsic to all cellular organisms assuring genome stability during DNA replication and in response to a variety of internal and external environmental threats that can generate primary and secondary DSBs. Two categories of DSB repair, nonhomologous end-joining (NHEJ) and homology-driven recombinational repair (HDRR), have been characterized in eukaryotes, including humans. End-joining enables reconnection of DSB ends utilizing little or no homology between the ends, while HDRR provides accurate repair of broken or gapped regions. As originally envisioned for HDRR of ionizing radiation-induced random DSBs [1] (IR; γ-DSBs), the initial step involving 5′ to 3′ resection provides directionality to repair and opportunities for repair synthesis across a break following homologous strand-invasion interactions. The components of resection have been studied extensively in yeast and other organisms (see reviews [2]–[5]) and reconstituted *in vitro* [6]. *In vivo* studies in budding yeast using HO-induced DSBs have shown that following break recognition the subsequent resection is a two-step process [7], [8]. In the initiation step of resection, the Mre11-Rad50-Xrs2 (MRX) complex and Sae2 remove ∼50 to 100 bases of DNA from the 5′ end, after which Exo1 and the combined activities of Sgs1 helicase with Dna2 nuclease carry out long 5′ to 3′ resection. DSB recognition and subsequent resection could be influenced by the type of break, such as “dirty” or clean, and the presence of Ku, which can protect ends from processing in the absence of MRX or Sae2 [9], [10]. The disruption of resection has been linked to gross chromosomal rearrangements (GCR) [11], [12] and carcinogenesis [13], [14]. Also, long-lived resected DNA is especially vulnerable to spontaneous and induced mutagenesis [15], [16].

Intrinsic to studies of DSBs and their repair is the possibility of interaction between the ends and, for the case of recombination, interactions with other molecules. For example, are ends held together, is there coincident resection, and what are the steps in the interactions with homologous chromosomes or sister chromatids? In the absence of coincident resection of both ends of a DSB, single-end resection intermediates might lead to break-induced replication (BIR) or half-crossovers, which could result in loss of heterozygosity, translocations, and other gross chromosomal rearrangements (GCR). There are several reports of increased BIR and rearrangements when MRX, Sae2, or the Mre11 nuclease is absent [17]–[19]. Hence, MRX-mediated communication between the two ends of the same DSB might prevent translocations between broken chromosomes.

Previously, we and others showed that MRX is required *in vivo* to hold DSB ends together, based on single molecule analysis of each end of I-*Sce*I-induced chromosome breaks using different fluorescent probes near the DSB [20], [21] or a common probe distant from the two sides of a DSB [22]. Tethering of defined DSBs by MR (Mre11/Rad50) complex, which has been described in crystal structures [23] and is observed *in vitro* [24], suggests that this complex could facilitate a “co-processing” mechanism of coordinated end-processing that would protect the genome from 1-end resection events. Alternatively, this function could also be accomplished by highly efficient independent processing of the two ends so that any 1-end resection intermediates would be too short-lived to pose much risk to genome stability. Here, we use the broader term “coincident resection” to include both of these models for processing both ends of DSBs.

Except for DSBs developed in meiosis [25] and interpretations derived from crystal structures, there has been no information about coordination or even coincidence of events between DSB ends. We sought to address the extent to which resection of both ends of random, IR-induced dirty DSBs in yeast might be coincident and the roles of the MRX complex and the genetically associated factor Sae2 [2]. The Sae2/MRX proteins are important for initiation and processing of “clean” enzymatically induced DSB ends, DSBs associated with inverted repeats, as well as Spo11 bound DSB ends in meiosis (reviewed in [2]).The Sae2/MRX proteins can also act at damaged single-strand ends based on survival studies in budding yeast [9] with the topoisomerase 1 inhibitor camptothecin and *in vitro* studies with covalently linked 3′-phosphotyrosine [26]. Using fluorescence imaging, Lisby et al [27] showed that in *Δsae2* and Mre11 nuclease deficient budding yeast mutants, Mre11 foci persist longer at IR-induced DSBs than at an I-*Sce*I-induced break, suggesting that these factors play a role in resection of damaged ends. Defects in the corresponding human and mice proteins increase cancer susceptibility [28]. Although lack of Mre11 nuclease is embryonic lethal in mice [29], specific roles for the budding yeast Mre11 nuclease have only been ascertained during meiosis (see review [30]) or in association with other defects (such as Ku70/80 endjoining proteins and Sgs1 [9], [10], [31]). In growing fission yeast, the Mre11 nuclease plays an important role in DSB repair but is not required for resection at an HO-induced DSB [32].

**Figure 1:**
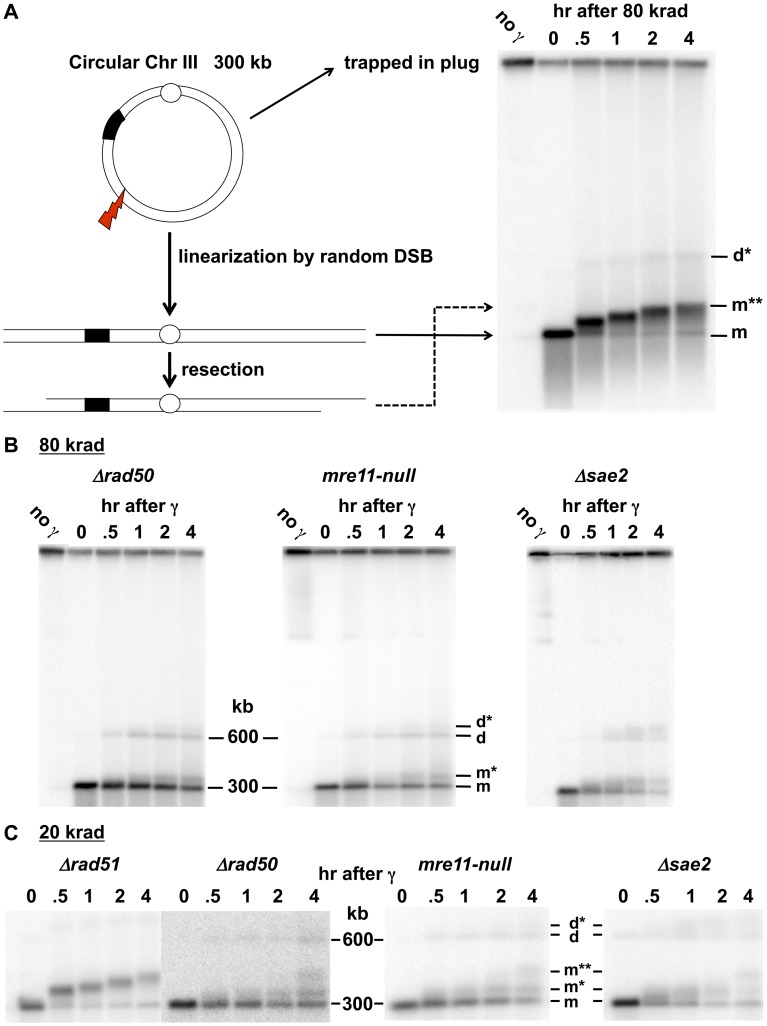
Resection at IR-induced single breaks in circular Chr III. A. Scheme for addressing γ-radiation induced DSBs and resection using circular chromosomes and PFGE-shift. As previously described in Westmoreland et al [33], the DNA of a circular Chr III is trapped in the well. A single DSB will result in a unit size linear molecule detected with a Chr III specific probe as a narrow band. A smear of smaller molecules under the full length Chr III monomer is due to multiple DSBs. Resection results in reduced mobility, referred to as “PFGE-shift.” B. & C. Circular chromosome based resection assay for random DSBs. Southern blot analysis of Chr III from cells arrested in G2 that were exposed to 80 krad (1B) and 20 krad (1C). Most of the IR linearized molecules (“m”, 300 kb) obtained from the *Δrad51* cells exhibit pulse-field shift within 30 min with a maximum shift by 4 hr. For *Δrad50* and *mre11-null*, most molecules remain unshifted even at 2 hr. Based on results below in Figure 2, the m* and m** (see 1C) correspond to 1- and 2-end resected molecules, respectively. The d and d* (shown in B and C) correspond to dimers due to recombination and resected dimers, respectively; they are greatly reduced in the *Δrad51* mutant (Figure 1A; discussed in [33]). The molecules that are detected in the upper part of the gel in 1B in some experiments are likely supercoiled [52]. The gels of *Δrad51* (Figure 1A) and *Δrad50* in Figure 1B and 1C are from [33]).

Before the present study it was not feasible to address *directly* resection at both ends of a DSB, or to distinguish 1- vs 2-end resections at random, radiation-induced dirty DSBs or even enzyme-induced clean DSBs. Previously, we reported that resection of broken chromosomes in budding yeast reduces pulsed-field gel electrophoretic mobility (“PFGE-shift”) and suggested that partial-shift bands observed in MRX-null strains might be due to uncoordinated, 1-end resections [33], [34]. Here, using an I-*Sce*I-induced DSB and extending PFGE analysis to a second PFGE dimension (see Figure 1A) we demonstrate unique patterns associated with broken chromosome molecules that have 0-, 1- and 2-end resections. This has allowed us to examine the roles that Sae2/MRX, and Mre11 nuclease play in the coincident, rapid initiation of resection at both ends of IR-induced DSBs.

## Results

### Rapid Initiation of Resection at IR-Induced DSBs Requires MRX

The PFGE-shift assay (see Figure 1A) provides a robust assessment of the timing and extent of resection at random DSBs induced by IR in circular chromosomes since all chromosomes with a single DSB are visualized as a population of linearized, unit length molecules [33]. In our previous work [33], some of which is included in Figure 1, we observed a rapid and nearly synchronous initiation of resection at IR-induced DSBs shortly after irradiation of **Δ** *rad52* and **Δ** *rad51* strains, which lack steps in HDRR following resection, as well as WT. The narrowness of the shifted bands suggests that there is little variation in timing or extent of resection. However, as shown between these two studies (Figure 1 and [33]), initiation of resection is severely delayed in the *mre11-null* and *Δrad50* mutants based on the persistence of unresected (*i.e.*, unshifted) molecules (designated “m” in Figure 1C). In addition, many molecules only exhibited a partial shift (m* in Figure 1C) with few molecules reaching the fully shifted position (m**) found at much earlier times in WT, *Δrad51* and *Δrad52* strains. The altered pattern of resection was not due to binding by Ku, as shown in Figure S1. (Note: The “d” and “d*” bands seen in Figure 1 were concluded to be unresected and resected, respectively, linear dimers of Chr III that resulted from recombination based on the requirements for Rad51 and Rad52, as discussed in [33].)

### 2-D PFGE Analysis Distinguishes Molecules with 0-, 1-, and 2- End Resection

**Figure 2:**
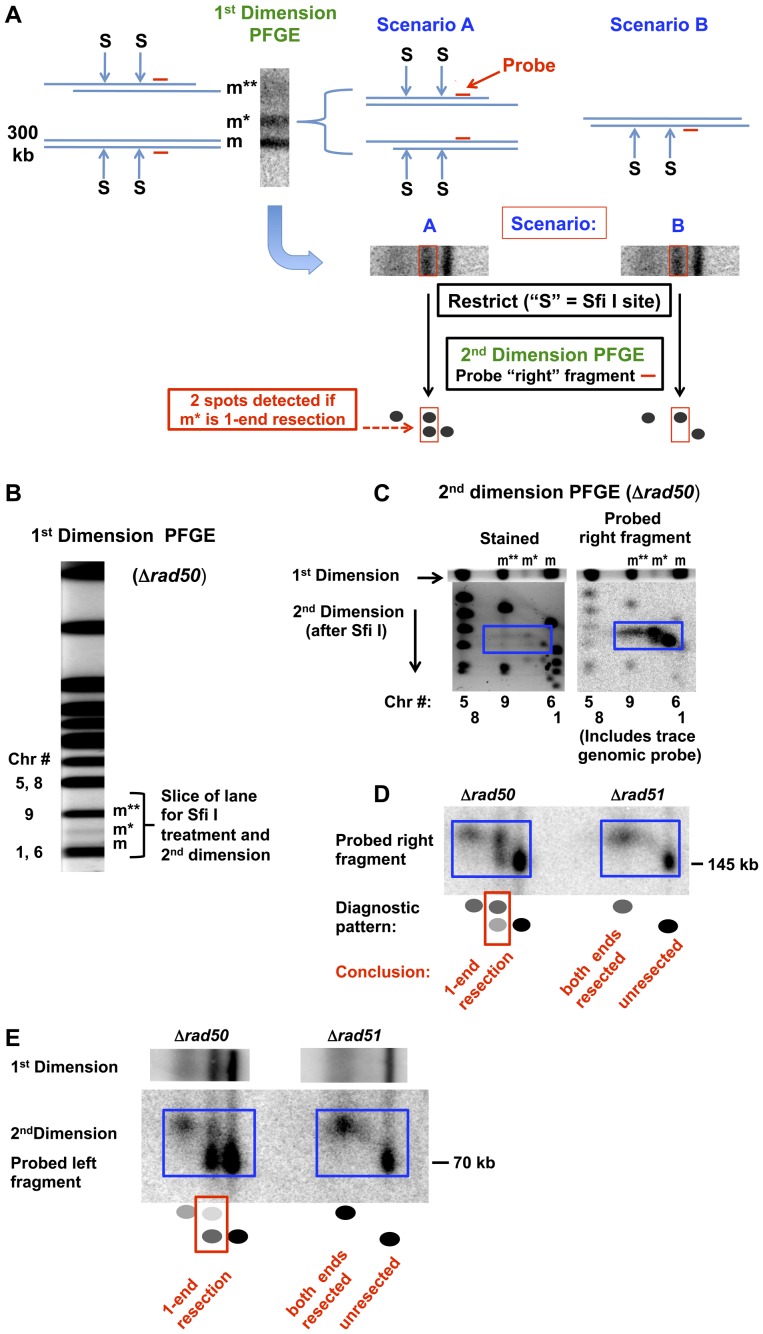
Detection of 1-end and 2-end resections at a DSB based on rare restriction cutter analysis of 1D PFGE-shifted molecules and subsequent 2D-PFGE. A. Diagram of approach to determine whether the m* and m** PFGE-shift bands of the broken Chr III are due to 1-end or 2-end resections. Cells are induced for I-*Sce*I, creating a single DSB in Chr III. The m band shown at the left of the gel-slice corresponds to a linear molecule with unresected DSB ends, and the m** band is proposed to be due to 2-end resections. The diagrammed molecules on the right of the gel slice represent two scenarios of resection that could account for the partial shift m* band. In scenario A molecules have extensive resection at only one or the other end while in scenario B both ends of the broken molecules have undergone only a small amount of resection. Diagrammed in the lower part of (A) is the subsequent treatment of the gel slice with the rare cutter SfiI (“S”), followed by PFGE in a second dimension and probing for only the right fragment, identified by a horizontal short red line. The m and m** bands containing molecules with 0- or 2-ends resected, respectively, would give rise to single spots. Scenario A predicts 2 spots for the m*, corresponding to molecules with no “right end” resection and those with a resected “right end.” Scenario B would only yield molecules with the right end resected. B. Stained PFGE gel (SYBR Gold, Invitrogen) of DNA from *Δrad50* cells that are induced for I-*Sce*I for 6 hr. The region of the lane containing the m, m* and m** molecules is indicated. C. Second dimension PFGE analysis of *Δrad50* using SYBR Gold stain and probe to “right” fragment. An unstained gel slice (“1^st^ dimension”) was equilibrated in TE (10 mM Tris, pH 8, 1 mM EDTA), treated with SfiI (New England Biolabs; 400 units in 4 ml total reaction volume including gel slice) for 5 hr at 50°C, followed by overnight treatment in 1% sarkosyl and 1 mg/ml proteinase K at 37°C and subsequent equilibration with running buffer and run in the second dimension. The left image corresponds to the SYBR Gold stained gel. The 3 forms (m**, m* and m) of I-*Sce*I linearized circle Chr III are identified above and the positions of other chromosomes are identified under the image. In the right image, a Southern transfer of the same gel is probed with DNA that is specific to the right end (also see Figure S2A) along with a small amount of labeled genomic probe to identify chromosome fragment positions on the gel. Note that in the SYBR Gold stained gel, the SfiI fragments of the m* band (in the blue box) are well-separated, indicating two distinct populations of the probed “right fragments.” D. Second dimension PFGE analysis of *Δrad50* and *Δrad51* using pure “right” fragment probe. The probe pattern for *Δrad50* corresponds to 1-end resections, as described in scenario A of (A), while the *Δrad51* pattern reveals resection at both ends of a DSB but no 1-end resections. E. Resection of the “left” side of the in vivo I-SceI cut chromosome III. The scheme described in (A) was followed except that the probe was specific for the left end of the I-SceI break. For *Δrad50* the m* position yields both unshifted and shifted molecules after SfiI digestion, consistent with 1-end resection as described for the right end in (D). There is a bias in 1-end resection among the m* molecules in that the “right” sides of the DSBs are more frequently resected than the “left” sides (also see Figure 3 and Figure S3). Note that for *Δrad51* there are no molecules at the m* position, which corresponds to 1-end resection. Similar results were found in another experiment that compared *Δrad50* and *Δrad52* (see Figure S3).

We anticipated that an understanding of the nature of shifted and unshifted molecules would provide insight into the underlying resection events and the roles of MRX as well as the Sae2 protein in coincident resection. Based on results with a model bacteriophage lambda system [33], we proposed that the partial shift molecules (m* in Figure 1B and 1C) seen in the MRX-null mutants might arise from MRX-independent initiation of resection at only one side of a DSB by other nucleases [4], such as Exo1 and Sgs1/Dna2. Alternatively, the molecules in the partial shift band might consist of short 2-end resections (see scenarios A and B, respectively, in Figure 2A). The rapidly appearing m** band obtained from the WT, *Δrad51* and *Δrad52* strains was proposed to be due to efficient, coincident/coordinated resection from both ends by the MRX complex. The architecture of the MR components accommodates both tethering of ends and resection [23] (see model described below in Discussion) that could assure coordination. However, apparent coordination might be accomplished by coincident, highly efficient but independent processing of the two ends. Our findings below establish coincident resection, which because of the structural features of MRX suggests coordinated resection, as presented in the Discussion.

To understand the pattern of shifted molecules, we examined events at a single defined break produced *in vivo* by I-*Sce*I and extended our PFGE analysis of resection to a second dimension (*i.e.*, 2-D PFGE). We reasoned that a broken circular chromosome provides the opportunity to directly address coincident resection, since both ends of a DSB are present on a single molecule (see Figure 1A). As diagramed in Figure S2A and described in Figure 2A, the approach involves cutting out the portion of the first dimension lane containing all three monomer forms of the *in vivo*, I-*Sce*I cut circular Chr III (m, m*, and m**) followed by SfiI restriction enzyme cutting of the PFGE-shifted and unshifted molecules before the 2nd dimension electrophoresis. If the m* band in the first dimension PFGE is composed of 1-end resected molecules (scenario A of Figure 2A), then SfiI digestion before the 2^nd^ dimension should give rise to two bands in the second dimension PFGE: shifted (resected) and unshifted (not resected) molecules. Alternatively, if the m* band is due to short but coincident resections at both ends of the I-*Sce*I break (scenario B of Figure 2A), then SfiI digestion should yield only shifted fragments in the 2^nd^ dimension.

**Figure 3:**
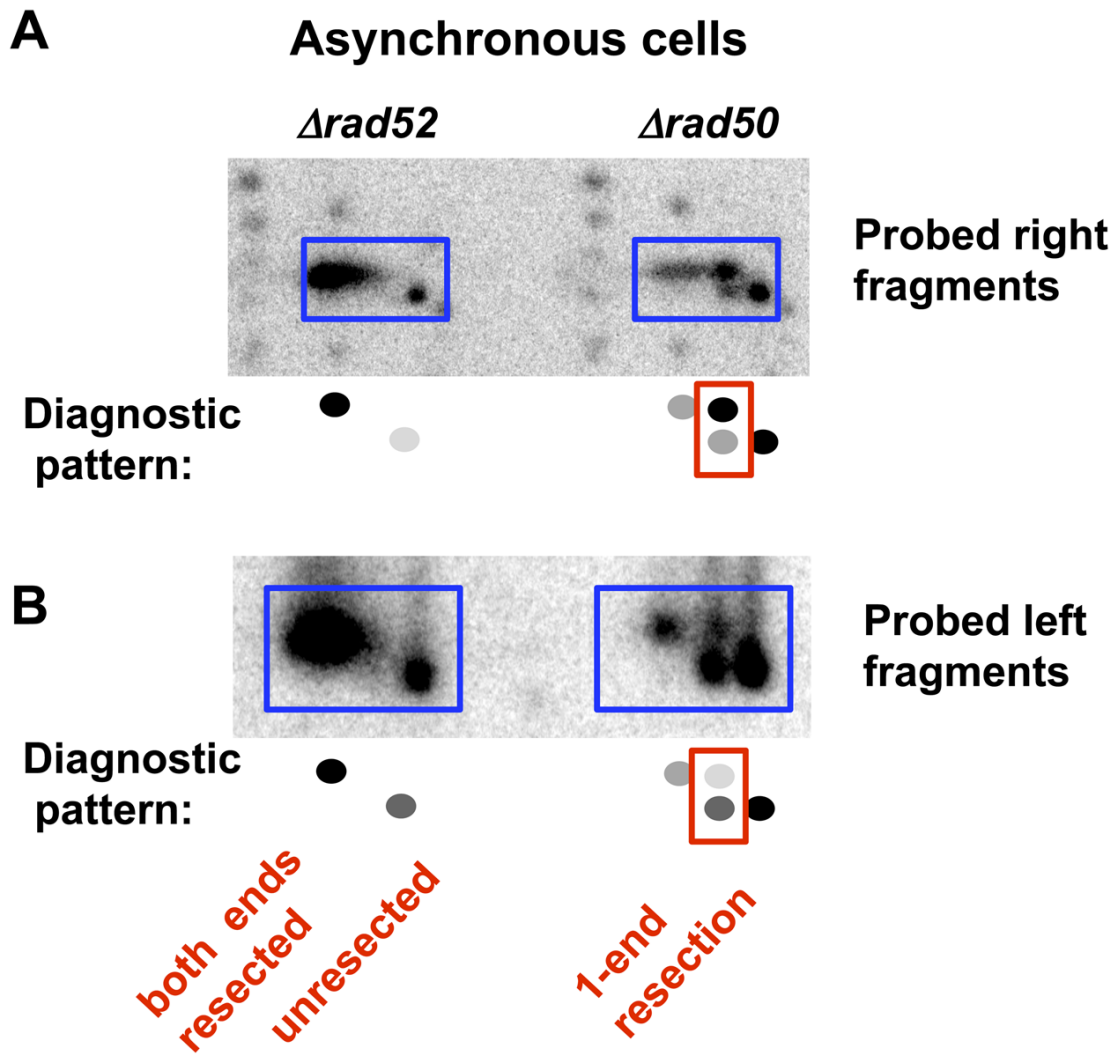
Resection at an I-*Sce*I-induced DSB in growing (asynchronous) *Δrad52* and *Δrad50* cells. 2-D PFGE analysis of resection of the right and left fragments after SfiI cutting. A. Right end probe (along with a small amount of labeled genomic probe to identify chromosome fragment positions on the gel). B. Left end probe (pure probe).

Presented in Figure 2 are the linearized Chr III molecules probed at sequences to the “right end” (2C and D) and the “left end” (Figure 2E and Figure S3) of the DSB following 6 hr induction of I-*Sce*I cutting. Approximately half the molecules obtained from the *Δrad50* mutant were at the “m” position, corresponding to I-*Sce*I breaks that were not resected at either end. About 30–40% were at the partially shifted m* position and the remaining were at m**. Based on the (SfiI+2D/PFGE) scheme of Figure 2A, the m* band is largely composed of molecules lacking resection at one end (scenario A). That is, the lower unresected spot under m* in Figure 2C and 2D migrates to a position comparable to the unresected I-*Sce*I cut molecules (the “m” band). Comparable results were found for asynchronous, growing cells (Figure 3). While the MRX complex is important in initiation of resection [3], [8], [33], our finding of 1-end resected molecules demonstrates that it is also required for coincident resection, even at a “clean,” I-*Sce*I-induced DSB.

Interestingly, there appears to be a bias in the end that is resected in the *Δrad50* cells. For example, when the left fragment is probed after (SfiI+2D/PFGE) the lower spot is stronger than the upper spot for both G2 (Figure 2E and Figure S3) and asynchronous (Figure 3B) cells. Conversely, when the right probe is used (Figure 2C and Figure 3A) the upper spot is more intense than the lower spot. Therefore, the 1-ended resections seen in the m* band are more often due to resection at the right side of the I-*Sce*I break than resection at the left side. There are several explanations for this bias that would be interesting to pursue, including sequence, chromatin, and differential binding of Ku or other proteins to the ends. However, regardless of the bias, both types of 1-ended resections are easily detected in the absence of MRX.

It should also be noted that Perrin et al [35] showed that *in vitro*, I-*Sce*I exhibits persistent strong binding affinity for the downstream side of the I-*Sce*I recognition site even after cutting. This corresponds to the right side of the I-*Sce*I DSB in our system. Such binding could inhibit MRX-independent initiation at the right side of the I-*Sce*I break. This could account for the portion of 1-end resections that are resected only at the left end, but could not account for the relatively higher number of right end only resection events.

We also tested the role of end-binding/end-joining Ku70 protein in MRX-independent initiation of resection at the I-*Sce*I-induced DSB and found that in a *Δrad50* *Δku70* double mutant, both types of 1-ended resections are easily detected as shown in Figure S4. Thus, even in the absence of Ku binding, coincident initiation of resection is highly dependent on MRX. There may be an increase in MRX-independent resection in the absence of Ku70, as suggested in other studies using HO, which cuts more efficiently than I-*Sce*I [10]. Furthermore, there is no apparent bias to resect the right side of the I-*Sce*I break as there is in the *Δrad50* single mutant (Figure S4).

Thus, following I-*Sce*I treatment of G2-arrested *Δrad50* cells, many of the linearized molecules experienced 1-end resection, demonstrating a strong requirement for MRX for coincident initiation of resection in budding yeast. Furthermore, the 1-end resections observed in *Δrad50* cells are largely independent of Ku binding and cannot be attributed to persistent I-*Sce*I binding to the right side of the break after cutting. Since the m** band gave rise to only a single band following SfiI+2D-PFGE, the molecules in this band were due to resection at both ends.

### Resection in *Δrad51* and *Δrad52* Strains Is Coincident

Unlike for *Δrad50*, “m*” molecules were not detected in the resection-proficient *Δrad52* and *Δrad51* strains following I-*Sce*I induction and 6 hr incubation (Figure 2D, right probe, and Figure 2E and Figure S3, left probe; and asynchronous cells, Figure 3). The molecules found under the m** position are all shifted in the second dimension, again confirming that the m** band is composed of molecules resected at both ends. We conclude that resection at both ends of an I-*Sce*I-induced DSB is coincident in the *Δrad52* and *Δrad51* strains. Surprisingly, a significant fraction of “m” molecules (*i.e.*, unresected) are found in *Δrad51* and *Δrad52* mutants (see Figure 2 and Figure 3, as well as Figure 5A below) unlike what was found for gamma-induced DSBs (Figure 1). Possibly the damage response system is less capable of detecting clean DSBs than dirty DSBs, or Ku complex binding is greater at clean DSB ends. Nevertheless, the detection of “m,” but not “m*,” molecules in these strains suggests that when MRX and Sae2 are both present, 1-end resection intermediates are either not present or are too short-lived to be detected in this assay.

The horizontal width of the m** band is likely due to variation in the extent of resection. Since DSBs are not induced synchronously or even rapidly in the culture by I*-Sce*I, the breaks that were induced later in the time course should have shorter resection tracks, resulting in some broken molecules having less than maximum PFGE shift in the first dimension.

### MRX Is Required for Coincident Resection at IR-Induced DSBs

Having established that m**, m* and m bands obtained in 1D-PFGE can be attributed to broken circular Chr III molecules that are resected at both ends, only one end, or neither end, respectively, we shifted our focus to the corresponding three bands seen after 1D-PFGE for IR-treated cultures. Since we never see an m* band in IR-treated *rad52*Δ or *rad51*Δ strains (Figure 1 and [33]), we conclude that, as for an I-*Sce*I-induced DSB, coincident resection predominates in these resection proficient strains.

In contrast to the *rad52*Δ or *rad51*Δ mutants, the very limited resection that occurs in the *rad50*Δ and *mre11-null* strains yields primarily m* molecules at earlier times demonstrating that MRX-independent initiation of resection at IR-induced DSBs is not only inefficient, but likely uncoordinated giving rise to 1-end resections. While this conclusion is based upon the above finding that m* corresponds to 1-end resections for I-*Sce*I induction of a break, it is formally possible that m* molecules following IR might arise through short 2-end resections, as described for Scenario B in Figure 2. However, the appearance of m* molecules in the *rad50*Δ and *mre11-null* following 20 and 80 krad and subsequent appearance of 2-end resected m** molecules after the lower dose (see Figure 1) renders Scenario B unlikely. The Scenario B explanation for the m* molecules implies coincident resection initiation at the 2-ends, after which the subsequent resection is prevented or at least inhibited from extending beyond the point corresponding to the m* shift position. However, Zhu et al and others [8], [10] demonstrated that for an HO-induced DSB in cells deficient in MRX, only the initiation step of resection is impaired; once resection is initiated, long resection proceeds at the wild type rate. Thus, we propose that the more parsimonious, 1-end resection model in Scenario A best explains the observed m* molecules at γ-DSBs, especially since it matches well with the results for the I-*Sce*I-induced DSBs.

### Sae2 and the Mre11 Nuclease Are Also Required for Coincident Resection at IR-Induced DSBs

The protein Sae2 (Ctp1) plays at least a limited role in initiation of resection of DSB ends in conjunction with MRX at defined HO-induced DSBs [7], [8], [36], [37]. Mimitou and Symington [9] have suggested that it might affect resection at dirty-end DSBs, possibly through an endonuclease activity [38], and its role in repair of radiation damage appears confined to S/G2 cells [39]. As shown in Figure 1B and 1C, the absence of Sae2 has consequences to resection at IR-induced DSBs comparable to mutations that completely abolish MRX function. The appearance of 1-end resected molecules (m*) is similar to that for the *Δrad50* and *mre11-null* mutant after 20 and 80 krad (Figure 1B and 1C). Thus, like the MRX complex, Sae2 is required for both efficient and coincident initiation of resection at γ-DSBs. However, the more rapid shift of broken Chr III molecules from the m to m* position in the *Δsae2* mutant indicates that the defect in initiation of resection is not as severe as in *Δrad50* or *mre11-null* strains.

**Figure 4:**
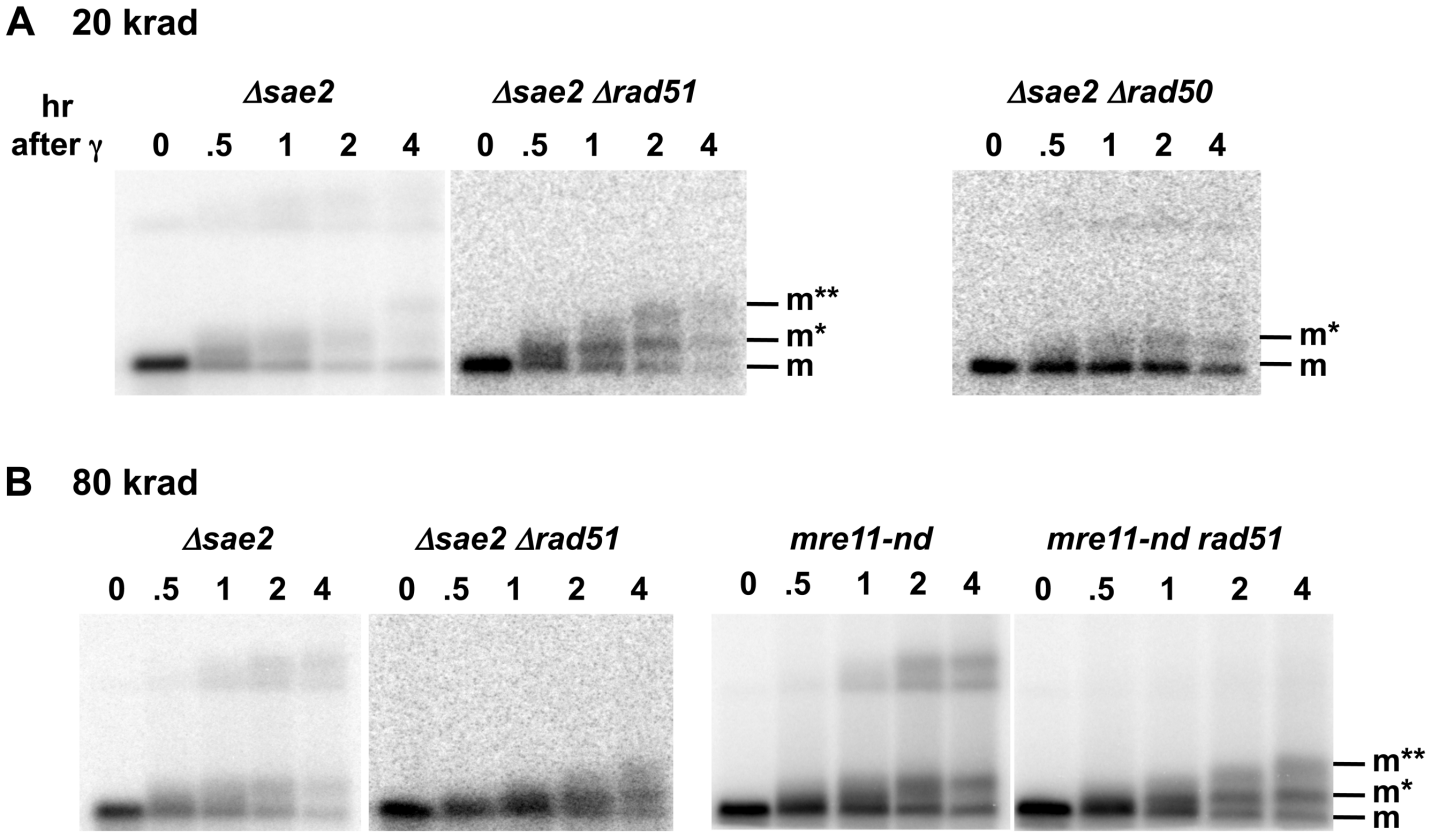
Sae2 and Mre11-nuclease are required for coincident resection, DSB repair, and high survival following IR of G2 arrested cells. Compared to the single mutant *Δsae2*, there is an increase in 1-end resected molecules and the appearance of 2-end resected molecules after IR treatment of the double mutant *Δsae2* *Δrad51*, which lacks the strand invasion step in HDRR. (The reduction in signal at 4 hr for *Δsae2* *Δrad51* in 3A is due to less DNA on the gel.) Thus, the loss of material at late times for *Δsae2* (Figure 1A and 1B) is likely due to HDRR. The response of the nuclease-defective *mre11-nd* and *mre11-nd* *Δrad51* strains (in 4B) is comparable to the *Δsae2* and *Δsae2* *Δrad51* mutants. The two upper bands seen in the *Δsae2* and *mre11-nd* single mutants correspond to unresected and resected forms of recombination dependent dimer molecules indicated as d and d* in Figure 1B and 1C. (Note: the *Δsae2* image in 4B is the same as in 1B and is presented here for comparison.) A. 20 krad. B. 80 krad.

**Figure 5:**
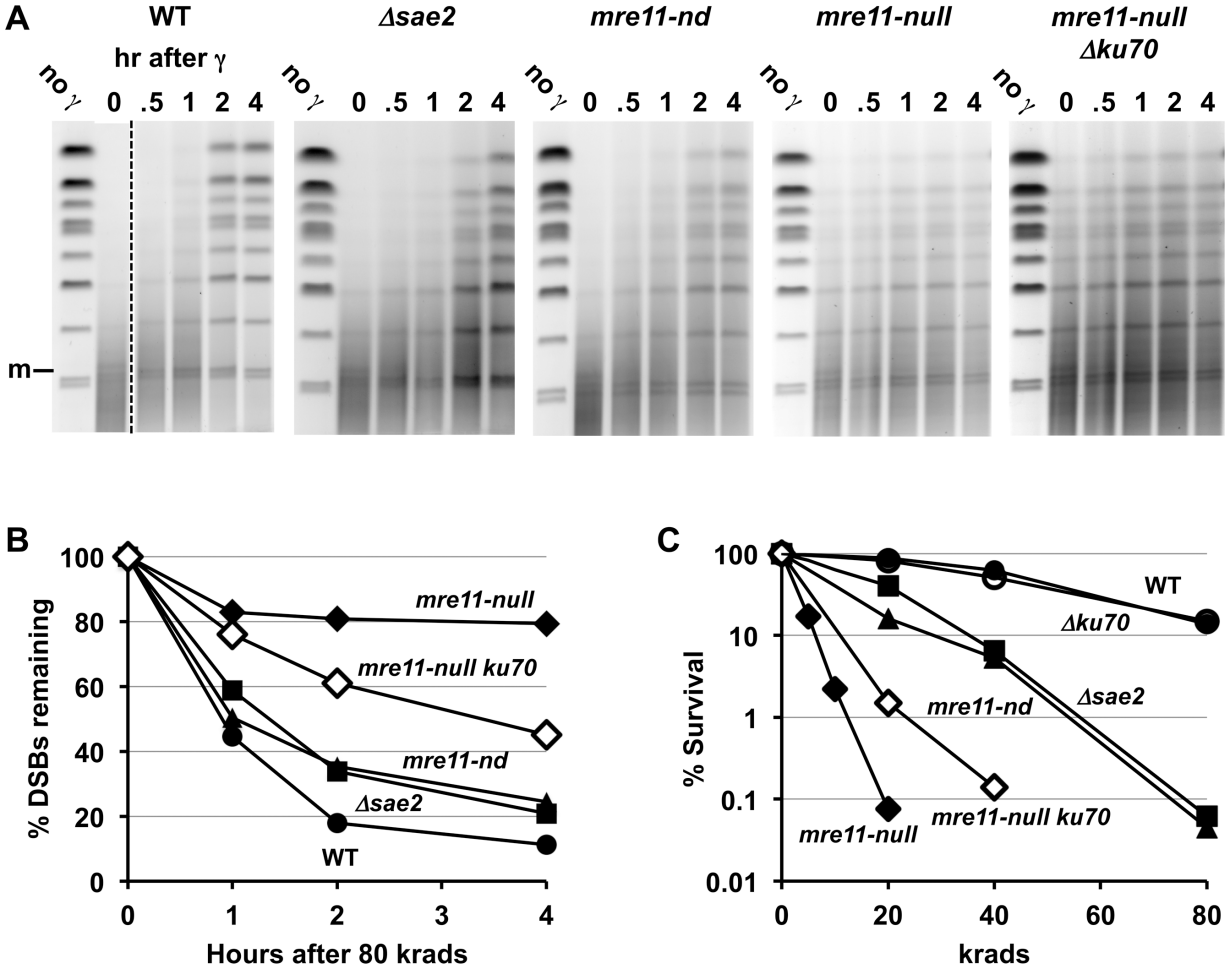
Impact of Δ*sae2* and *mre11-nd* mutations on repair of IR-induced DSBs. A. Genomic chromosomal changes in response to 80 krad and subsequent repair. Some of the lanes for the WT were removed to provide matching time course information between WT and mutants. Note the appearance of the broken circular chromosome (m) at time “0” after irradiation, just above the lowest two bands. The PFGE gels were stained with SYBR Gold to identify the DNA. B. Repair of DSBs following 80 krad. Quantitation of DSBs is derived from an analysis of broken chromosomes in the SYBR Gold stained PFGE gels of (A). Analysis of the frequency of DSBs and repair are described in [33]. WT, circle; *Δsae2*, square; *mre11-nd*, triangle; *mre11-null*, diamond; *mre11-null* *Δku70*, open diamond. C. Survival of irradiated cells. Following irradiation, cells were diluted and plated to YEPD and survival was determined based on number of colonies. WT, circle; *Δku70*, open circle; *Δsae2*, square; *mre11-nd*, triangle; *mre11-null*, diamond; *mre11-null* *Δku70*, open diamond.

It is interesting that when later steps in HDRR were blocked in the *Δsae2* *Δrad51* double mutant there was a substantial increase in the proportion of 2-end resected molecules seen above the m* position. This was especially evident by 1 and 2 hr after 20 krads (Figure 4A) but also clearly evident at 2 and 4 hr after 80 krads (Figure 4B). The lack of 2-end resected molecules in the *Δsae2* single mutant relative to the *Δsae2* *Δrad51* double mutant is likely due to HDRR mediated repair, which could occur rapidly after initiation of resection of the second end. After repair, the re-circularized Chr III can no longer enter the gel, consistent with less material in the lane. This interpretation is supported by the DSB repair and gamma survival results, which are discussed below and presented in Figure 5.

The Mre11 nuclease, which has endonuclease and 3′ to 5′ exonuclease activity, is considered a likely candidate for resection at dirty DSB ends. In meiosis, 5′ to 3′ resection at a Spo11 protein blocked end appears to occur through both the endonuclease and 3′ to 5′ exonuclease activities of Mre11. These activities along with Exo1 can generate 5′ to 3′ resection [40]. Although several Mre11 active-site nuclease mutants have been examined, they have at most a small effect on resection at defined DSBs in vegetative cells [9], [41]. They also exhibit a modest effect on gamma survival even for those cases where the ability to form complex has been demonstrated, such as *mre11-H125N* (referred to in this study as *mre11-nd*) [42], but no effect on chromosome tethering at a chromosome break [20] (for *mre11-nd* and *mre11-D16N*). Significantly, unlike what has been reported for HO breaks, the *mre11-nd* mutant had a dramatic impact on resection of IR-induced DSBs, comparable to that of the *Δsae2* mutant, as shown in Figure 4b. Like *Δsae2*, the PFGE shift pattern of the *mre11-nd* mutant resembled that of MRX-null mutants far more than the pattern for *Δrad51* or *Δrad52* (Figure 1 and Figure 4). The persistence of unshifted “m” molecules indicates an overall defect in initiation of resection, and the existence of “m*” molecules demonstrates a lack of coincident initiation of resection in the absence of a functional Mre11 nuclease. Finally, the apparent lack of 2-end events, except when HDRR is inactivated in the double mutant *mre11-nd Δrad51*, supports the interpretation that repair occurs very quickly after the 2^nd^ end resection is initiated, as suggested above for *Δsae2*.

### Loss of Sae2 and the Mre11 Nuclease Decreases γ-DSB Repair and IR-Survival

The striking impact that the various mutants have on resection in the first 1–4 hr after IR correlates with DSB repair and survival. As summarized in Figure 5B, we show for the first time a direct measure of the impact of the *Δsae2* and *mre11-nd* mutants on DSB repair in real time: there is a modest ∼20–30% decrease in the rate of repair in the *Δsae2* and the *mre11-nd* mutants compared to WT for DSBs after 80 krad (∼120 DSBs per cell [33]). At 4 hr there are about twice as many DSBs remaining, whereas there is little repair in the *mre11-null* (Figure 5B) or the *Δrad50* mutant [33]. As shown in Figure 5C, the absence of either Sae2 or Mre11 nuclease activity increased IR sensitivity about 2-fold (*i.e.*, the dose modifying factor that yields survival comparable to WT), whereas sensitivity is at least 10- to 20-fold greater for MRX-null, *Δrad51* and *Δrad52* mutants.

### Loss of Ku70 Partially Supresses γ-DSB Repair and IR-Survival Defects of mre11-null

The severe DSB repair defect found in the *mre11-null* mutant was partly alleviated in an *mre11-null* *Δku70* double mutant (Figure 5B). The repair efficiency was intermediate between that of the *mre11-null* and either *Δsae2* or *mre11-nd* mutants since the %DSBs remaining in the double mutant at 2 and 4 hours was roughly twice that of *Δsae2* or *mre11-nd*. These results correlate with the modest increase in IR-resistance seen in *mre11-null Δku70 cells as compared to an mre11-null single mutant (Figure 5C).*

### Sae2 and the Mre11-Nuclease Have a Reduced Role in Resection at Clean DSBs

**Figure 6:**
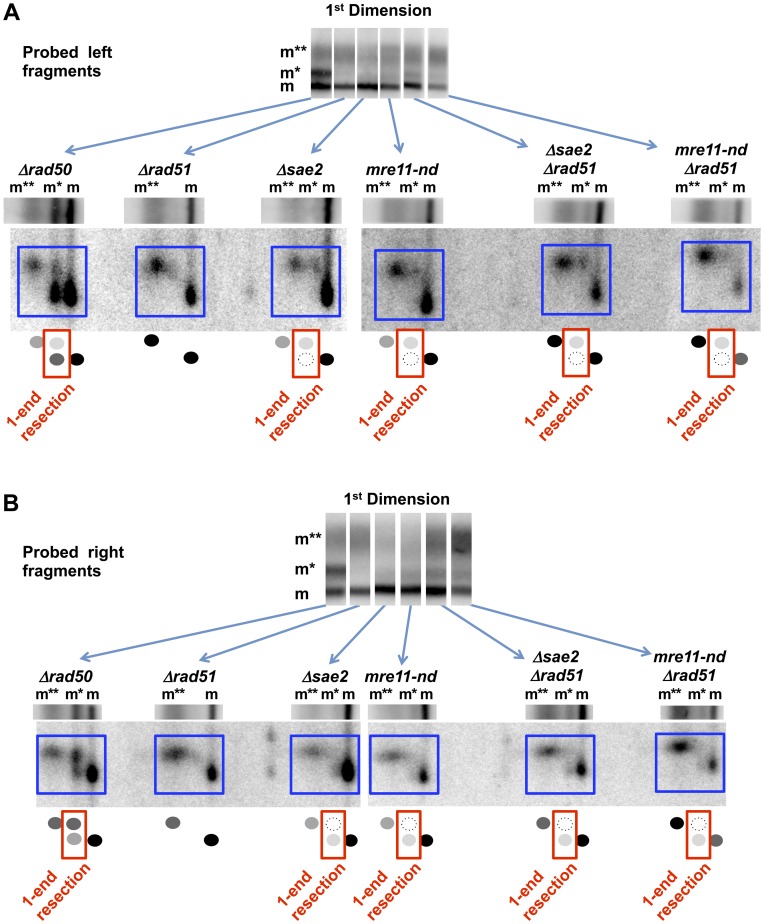
Resection at an I-*Sce*I DSB in *Δsae2, mre11-nd*, *Δrad50*, and *Δrad51* single and double mutants. Resection at a “clean” I-*Sce*I DSB in the G2 *Δsae2* and *mre11-nd* cells is primarily 2-ended regardless of the presence of the *Δrad51* mutation, unlike for γ-DSBs (see Figure 1 and Figure 4). However, resection at an I-*Sce*I break in the *Δrad50* mutant is primarily 1-ended (also see Figure 2). The small amount of 1-end resected molecules (m*) in the *Δsae2* and *mre11-nd* mutants is biased to resection at the left side of the break, while the 1-end resected molecules in the *Δrad50* mutant are biased to the right side. A. Left end probe. Note: *Δrad50* and *Δrad51* are also included in Figure 2E and are presented here for comparison. B. Right end probe. Note: *Δrad50* and *Δrad51* are also included in Figure 2D and are presented here for comparison.

Unlike for γ-DSBs, there was little impact of the *Δsae2* and the *mre11-nd* mutations on resection at an I-*Sce*I-induced “clean” DSB as shown in Figure 6A and 6B (left-end and right-end probes, respectively) based on the appearance of mostly m** or m bands of broken molecules after 1D-PFGE or (SfiI+2D/PFGE) analyses. Overall, the 2-D PFGE shift patterns shown in Figure 6 resemble those of *Δrad51* and *Δrad52* far more than *Δrad50*. Nonetheless, the presence of a small amount of 1-end resection intermediates (m* position) for both *Δsae2* and *mre11-nd* indicates that some resection at the two ends of DSBs is not coincident even at a clean, I-*Sce*I-induced break. These results demonstrate that Sae2 and the Mre11-nuclease play a limited role at clean breaks unlike the strong requirement for them in the processing of IR-induced breaks.

Surprisingly, among the “m*,” 1-end resected molecules in the *Δsae2* and *mre11-nd* mutants, there appears to be a bias to initiate resection at the left end. When the right fragment was probed (Figure 6B), most of the label under the 1^st^ dimension “m*” position was seen in the lower, unshifted position in the second dimension. The opposite is true for the left fragment probe (Figure 6A). This is the reverse of the bias that was seen in the *Δrad50* mutant. It is possible that the small portion of molecules that were resected only on the left side is due to persistent I-*Sce*I binding to the right side of the recognition site after cutting as discussed above (and see [35]).

## Discussion

Given the beneficial and damaging impact of DSBs in a variety of chromosomal processes, genome stability and disease, it is important to characterize their processing and consequences. The present study extends our understanding of some of the earliest events in DSB repair to the issue of coincident end-processing. Coincidence could assure accurate HDRR of DSBs in addition to rapid repair. Defects in the initiation step might be expected to result in long-lived, 1-end resection intermediates that could lead to genome instability resulting, for example, from BIR and/or channeling the individual ends into different repair pathways (*i.e.*, endjoining and BIR). Using the PFGE approaches that we developed, we establish that not only the MRX complex, but also Sae2 and Mre11-nuclease are required for coincident-resection of IR-induced DSBs.

Furthermore, the requirement for MRX in coincident resection of IR-or I-*Sce*I-induced DSBs is independent of Ku.

### PFGE-Shift Patterns Can Be Used to Determine Resection Events and Coincidence at DSBs

Previously, we concluded that MRX is essential to efficient initiation of resection of radiation-induced DSBs based on the 1D PFGE-shift patterns [33]. For all strains examined, there was an initial band (m) of linearized molecules immediately after irradiation that was due to molecules with unprocessed ends. In the wild type, as well as *Δrad51* and *Δrad52* strains, molecules shifted as a band (m**) and were concluded to be due to resection. This contrasted with *Δrad50 and mre11-null* where most molecules remained at the unshifted (m) position, indicating a general failure to initiate resection. Among the molecules that were resected in the absence of MRX, most shifted to an intermediate position (m*). Although it is formally possible that other single-strand lesions might impact resection and even lead to gaps, we consider this as an unlikely source of differences between the mutants. In addition, treatment of the DNAs with mung bean nuclease [33] did not lead to dramatic differences in the size of the DNA, which would have occurred had there been gaps. Using the 2D-PFGE approach developed in this study to examine events at a defined DSB, we have now confirmed that molecules in the m* band result from resection at only 1-end and that resection in the m** molecules is 2-ended.

Based on the appearance of m, m* and m** molecules, we could identify resection properties of the various mutants at IR-induced DSBs in order to address the issue of coincidence of resection at the two ends of DSBs. Since the m* class of molecules is due to resection at only one end of a DSB, MRX-independent resection is concluded to lack coordination. This contrasts with WT, *Δrad51* and *Δrad52* where all detectable resected molecules are resected at both ends (m**). The absence of m* demonstrates that functional MRX and Sae2 ensures coincident initiation of resection.

### The Impact of Sae2 and Mre11-Nuclease on Resection Is Greater at Dirty Versus Clean Breaks

We establish that defects in Sae2 and the Mre11 nuclease have a dramatic impact on the coincident initiation of resection at IR-induced DSBs similar to loss of MRX function. There is a gradual accumulation of 1-end resected molecules and almost no 2-end resected molecules (Figure 1 and Figure 4). However, m** molecules are detected when the Sae2 and the Mre11 nuclease mutants are combined with *Δrad51*, which lacks later steps in recombinational repair (Figure 4B). This suggests that DSB repair can occur in the single mutants soon after the initiation of resection of the 2^nd^ end of the γ-DSB and that initiation of resection of the second end is the rate-limiting step for DSB repair in *Δsae2* and *mre11-nd* single mutant strains. These results are consistent with the observed increase in DSB repair and survival in the single Sae2 and the Mre11 nuclease mutants compared to MRX null mutants (Figure 5B and 5C) as well as a recombination execution checkpoint, as suggested by Jain et al [43].

However, unlike the MRX complex, the Sae2 and Mre11-nuclease mutants have considerably less impact on clean I-*Sce*I-induced breaks. The stronger role for Sae2 and Mre11-nuclease at radiation-induced breaks suggests that they are necessary for the removal of at least a subset of damaged ends at the initiation step of resection, similar to the removal of Spo11 protein from the ends of meiotic DSBs (summarized in [40], [44]).

Possible differences in processing of dirty vs clean DSBs had been previously suggested although not directly demonstrated [9], [41]. A role for Mre11-nuclease and Sae2 at dirty DSB ends was postulated by Moreau et al [45] based on radiation sensitivity and supported by the findings of Lisby et al [27]. Employing elegant imaging approaches to address events in single cells, they [27] found that Mre11 foci were longer-lived in *Δsae2* or Mre11-nuclease deficient cells following 4 krad (corresponding to ∼8 DSBs per haploid G2 cell based on measurements of Westmoreland et al [33]) as compared to cells containing I-*Sce*I-induced clean DSBs. This is consistent with our direct molecular analysis showing that Sae2 and Mre11-nuclease play a much greater role in resection at dirty as compared to clean DSBs.

Alternatively, the increased requirement for Sae2 and Mre11-nuclease for resection of IR-induced DSBs might be due to the larger number of DSBs in the cell after 20 or 80 krads (40 or 160 DSBs per G2 cell compared to only 1 or 2 DSBs per G2 cell for an I-*Sce*I break). This possibility does not appear to be the case since there was comparable resection at a clean DSB in WT and Mre11 nuclease-deficient strains regardless of the presence of additional HO-induced DSBs [41].

In spite of the finding that resection in the absence of Sae2 or Mre11-nuclease was much less impaired at a clean I-*Sce*I-induced break than at IR-induced DSBs, the appearance of small amounts of m* molecules in the *Δsae2* and *mre11-nd* strains (Figure 6) indicates that a lack of coincident resection can occur in these mutants even at clean DSB ends. The structurally intact MRX complex contained in these mutants could lessen the requirement for a Sae2/Mre11 nuclease-mediated initiation step by tethering of the DSB ends and recruitment of the Exo1 and Sgs1-Dna2 nucleases to the break site.

For IR-induced DSBs some types of dirty ends may be resistant to initiation by either Exo1 or Sgs1, in which case recruitment of these factors by the MRX complex would not remove the requirement for initiation by Sae2 and Mre11 nuclease, hence the substantial m* bands (Figure 4A and 4B). On the other hand, for clean, enzymatically induced DSBs, MRX recruitment of these resection factors should enhance the ability of Exo1 or Sgs1 to initiate resection in the absence of Sae2 or Mre11 nuclease. This could occur by relatively efficient but independent initiation of the two ends by Exo1 or Sgs1-Dna2, which would result in most, but not all molecules initiating resection of the 2^nd^ end fairly soon after initiating the first end. The fraction of molecules in the m* band would depend on the average length of time between the first and second initiation events. Only molecules for which the first end experienced substantial long resection before the 2^nd^ end was initiated would migrate at the m* position in the first dimension.

### Conclusions and Implications

The architecture of the MR components accommodates both the tethering of ends along with a co-processing mechanism of resection through a single complex [23], [24]. The appearance of molecules with either no resection (m) or resection at both ends (m**), but no intermediate molecules with only 1-end resection following expression of I*-Sce*I in *Δrad51* and *Δrad52* mutants (Figure 2D and 2E, Figure 3A and 3B, and Figure S3), supports the view that coincident initiation of resection is coordinated via a single complex. However, it is formally possible that 1-end intermediates do exist in these strains, but are too short-lived to be detected in our PFGE-shift assay.

Coincident resection may enable rapid repair in mitotic cells [30 to 50% within 1 hr (see Figure 6 and [33]] as well as prevent 1-end genome destabilizing events such as BIR, which could lead to loss-of-heterozygosity (LOH; summarized in [46]) or prevent rearrangements via interactions of repeats, such as Ty elements [47]. (BIR appears to take much longer than the resection and repair events studied here [43], [46].) Interestingly, the absence of MRX, Mre11 nuclease, or Sae2 can lead to gross genomic changes including BIR at a unique DSB [17]–[19]. Previously, MRX complex was found to be required *in vivo* to efficiently hold DSB ends together based on single molecule analysis of each end of I-*Sce*I-induced chromosome breaks using two different color probes close to either DSB end [20], [21] or using single color probes at positions distant from an HO-induced DSB [22]. The specific impact of the Sae2 and Mre11 nuclease mutants on coincident resection is best explained by defects in initiation of resection at dirty γ–DSBs rather than a defect in tethering since neither Sae2 nor Mre11 nuclease is required to prevent chromosome breaks [20], [21].

**Figure 7:**
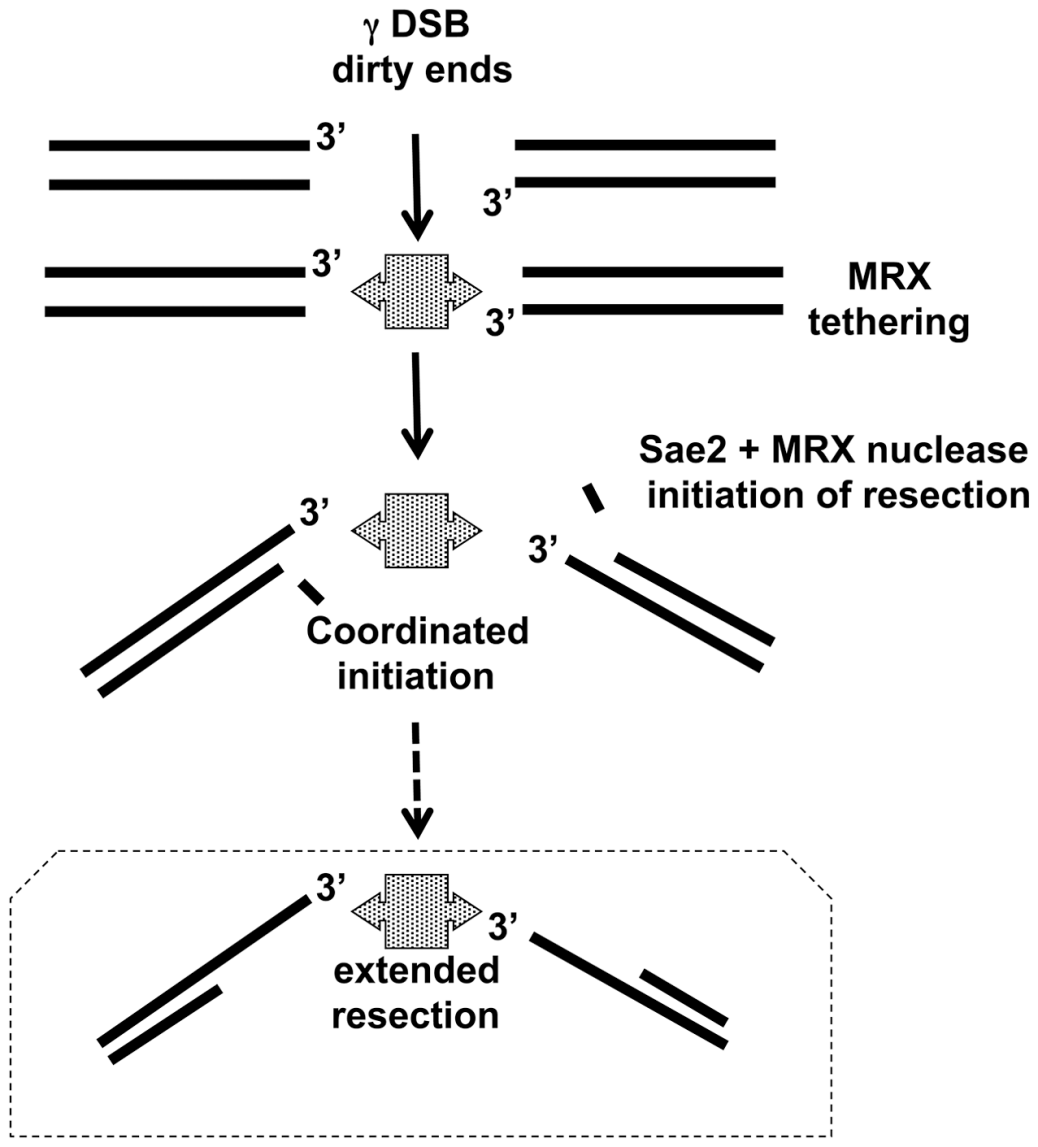
Model for coordination of resection. γ-radiation induced DSBs with dirty ends are tethered by MRX complex, which is consistent with MRX preventing a chromosome break at a clean I-*Sce*I cut site [20] and [21] as well as *in vitro* results [24]. In this model Sae2, MRX, as well as the Mre11 nuclease provide for coordinated resection. Subsequent extended resection of γ-radiation induced DSBs might be accomplished with Exo1 and/or Sgs1/DNA2 (addressed in [33] and unpublished). Note: In WT cells, ∼50% of IR-DSBs are repaired in 1 hr after 80 krads, during which time there is ∼1–2 kb resection per end.

In light of the structural information and the present results we propose the model described in Figure 7 to describe how Sae2/MRX coordinated resection might be accomplished through a combination of MR tethering and Sae2/Mre11-mediated initiation of resection at the dirty ends. After coordinated initiation and removal of dirty ends, the elongation step might be carried out by the Exo1 and/or Sgs1-DNA2 nucleases as shown for site-specific DSBs [7], [8]. The end-binding Ku proteins could influence pathways for resection of IR-induced DSBs when MRX is absent [9], [31]. However, they do not appear to be responsible for the lack of coincident resection at radiation or I-*Sce*I-induced DSBs in MRX-null mutants. Using the PFGE systems that we have developed, it will be interesting to explore the roles that Exo1 and/or Sgs1-Dna2, Ku and chromatin factors may play on resection at clean and dirty DSBs.

Overall, these findings reveal new roles for Sae2 and MRX, including the Mre11 nuclease, in processing of IR-induced DSBs and may provide insights into evolutionarily conserved DSB repair mechanisms in human cells. The Sae2 and MRX proteins are required for rapid and coincident resection of the damaged DSB ends, which contributes to repair and survival. These functions are likely to extend to other lesions with dirty ends, such as indirect DSBs [34] and those with bulky lesions, and have implications for agents used in chemotherapy.

## Materials and Methods

### Strains

All strains in this study are haploid, contain a circular Chr III and were derived from isogenic strains MWJ49 and MWJ50, described in [48]. Gene deletions in these strains were created by replacement of the relevant ORF(s) with dominant-resistance cassettes G418 (*kanMX4*), hygromycin (*hphMx4*), or nourseothricin (*natMX4*) as described in [49]. The I-*Sce*I recognition site and GAL1-I-*Sce*I gene were introduced into circular Chr III strains by integration of GAL1-I-*Sce*I core cassettes amplified from plasmids pGSHU or pGSKU [50] using primers

GSU_LC3 (5′ATCAAATTCGATGACTGGAAATTTTTTGTTAATTTCAGAGGTCGCCTGACGCTAGGGATAACAGGGTAATTTGGATGGACGCAAAGAAGT3′)

GNU-RC3 (5′ATGAAAAGCCGGTTCCGGCGCTCTCACCTTTCCTTTTTCTCCCAATTTTTCAGTTGAAAATTCGTACGCTGCAGGTCGAC3′).

The integration site is ∼250 bp to the left of the *LEU2* gene on Chr III. The *mre11-nd* mutation was introduced into circular Chr III strains by crossing with KS435 [20] followed by tetrad dissection and selection of appropriate markers. The *mre11-null* strain used in this study was made by integration of the KanMX-URA3core [51] into the *MRE11* locus. Primers

mre11-3 (AGTTCACAAGCAAGCCTGTA) and

mre11-4 (ACTTGTGAGGGATCGCTC)

were used to amplify the core cassette to enable targeting of the core cassette. The integration resulted in replacement by the core cassette of 485 bp of the *MRE11* locus beginning 215 bases upstream of the start codon through the first 169 bases of the coding sequence.

### Nocodazole Arrest, Gamma Irradiation, and Post-Irradiation Incubation

Nocodazole (United States Biological, Swampscott, MA) was used at 20 ug/ml final concentration in logarithmically growing cultures at 30°C in YPDA media (1% yeast extract, 2% Bacto-Peptone, 2% dextrose, 60 µg/ml adenine sulfate). G2 arrest was monitored by cell morphology. Cultures were harvested by centrifugation, washed and resuspended in ice-cold sterile water at ∼5×10^7^ cells/ml. Cell suspensions were kept on ice during irradiation in a ^137^Cs irradiator (J. L. Shepherd Model 431). Following irradiation, cells were centrifuged and resuspended in YPDA at 30°C and nocodazole was added at 20 ug/ml for post-irradiation incubation.

### Galactose Induction of In Vivo I-SceI Cutting

Cells were cultured logarithmically in YEP lactate as described in [33], and treated with nocodazole as described above for gamma irradiated cultures before adding 2% galactose.

### PFGE Procedures

Plug preparation of samples and PFGE using a CHEF Mapper XA system (Bio-Rad, Hercules, CA) were carried out as in [33] except for the details presented below that are specific for the 2D gels displayed in Figure 2, Figure 3, Figure 6, Figure S2B, Figure S3, and Figure S4.

Plugs were prepared using 0.35% LE (NOTE: not low melting) agarose (Lonza Allendale, NJ) and were formed in Beckman Geneline plug molds having dimensions 2.5×2.5×25 mm. The 1^st^ dimension CHEF gels used 0.75% SeaKem Gold agarose (Lonza) and were made using 3 mm thick plastic spacers sandwiched between the Biorad CHEF gel tray and a glass plate to give the gels a uniform thickness of only 3 mm. Uncut 2.5 cm long plugs were equilibrated in running buffer (0.5× TBE) before loading the entire plug in the gel (wide orientation of gel frame). The gels were then run for 22 hr using the auto-algorithm mode, programmed for 250 to 1400 kb.

Following the 1^st^ dimension run, the DNA-containing lanes were 2.5 cm wide (i.e., the plug width) and ∼13 cm long. To obtain material for the 2^nd^ dimension CHEF, two slices (∼4 mm×13 cm each) were cut and removed from the center of the 1^st^ dimension lanes (leaving about 8 mm to either side). The remaining flanks of each lane were stained with SYBR Gold, which enabled the bands of interest to be located. This procedure avoided the need to stain the lanes prior to the 2^nd^ run, since the stain interfered with the migration of chromosome fragments in the 2^nd^ dimension. After staining the flanks for 10 minutes in SYBR Gold, they were digitally photographed, and a ruler was used in the photograph to identify the region of the unstained lane slice corresponding to ∼220 kb to 600 kb (from just below Chr I to just above Chr V). This section was cut from the full-length lane slices and processed for the 2^nd^ dimension PFGE as follows: each lane section was equilibrated in 3 changes of 25 ml sterile TE (10 mM Tris, pH 8, 1 mM EDTA), treated with SfiI (New England Biolabs, Ipswich, MA; 400 units in 4 ml total reaction volume including gel slice) for 5 hr at 50°C, followed by overnight treatment in 1% sarkosyl (Sigma, St. Louis, MO) plus 1 mg/ml proteinase K (Invitrogen) at 37°C and subsequent equilibration with running buffer. The map of the positions of the SfiI restriction sites relative to the I-*Sce*I cut site are presented in Figure S2A and results for cutting the DNA by I-*Sce*I *in vitro* (prior to 1^st^ dimension) and SfiI (prior to 2^nd^ dimension) are presented in Figure S2B.

For the 2^nd^ dimension PFGE, the treated lane slices were placed near the top of the gel tray (long orientation of gel frame to allow for a long run) using an end plate from the casting stand on the anode side of the lane slices to stabilize the position of the slices so that they could be attached to the gel tray with 2 to 4 ml of agarose on the cathode side of the lane slices. After allowing the agarose to cool for 5 to 10 minutes, the end block was carefully removed, leaving the lane slices attached to the gel tray. The gel tray was then mounted in the casting stand, and 180 ml of 1.5% LE agarose (Lonza) in 0.5× TBE was carefully poured over the tray containing the attached lane slices. This was enough volume to cover the lane slices. The 2^nd^ dimension CHEF was run using the same autoalgorithm as the 1^st^ dimension, 250 to 1400 kb, except that a longer run time of 48 hr was used to compensate for slower electrophoretic mobility in 1.5% agarose.

### Southern Transfer Hybridization

Neutral Southern blots, probe preparations, ^32^P labeling and hybridizations were carried out as previously described [48]. Primers used for PCR amplification of genomic DNA to be used in the preparation of probes are listed in Table S1.

### Quantitation of DSBs

The DSB repair data shown in Figure 5B were calculated using the “stained gel, multiple band method” described in [33].

## Supporting Information

Figure S1 1-end resection of radiation-induced breaks in *mre11-null* *Δku70* cells. Presented are Southern blots of Chr III from cells arrested in G2 that were exposed to 80 krad and 20 krad. The PFGE-shift pattern for the *mre11-null* *Δku70* double mutant strongly resembles that of the *mre11-null* and *Δrad50* single mutants shown in Figure 1B and 1C. At both 80 and 20 krads, the presence m* molecules indicates 1-end resections, and fully shifted m** molecules are not detected after 80 krads. The WT and *Δku70* strains have PFGE shift kinetics similar to *Δrad51* (Figure 1A and 1C), indicating coincident resection at both sides of DSBs. Unlike *Δrad51*, these strains are repair proficient, so that the resected molecules diminish with time as they are recircularized by HDRR. After 20 krads, ∼80% of DSBs were repaired in the first hour (data not shown), so that the shifted band is very faint after 1 hour.
(TIF)

Figure S2 2D-PFGE analysis of I-*Sce*I cut site and SfiI restriction site targets in Chr III. A. Map of circular Chr III with I-*Sce*I cut site and restriction site targets of the rare-cutter SfiI. B. 2D-PFGE pattern after *in vitro* I-*Sce*I digest of plugs of unirradiated cells. An *in vitro* I-*Sce*I digest of the *Δrad50* strain containing an I*-Sce*I site shows the positions of the three I-*Sce*I/SfiI fragments of circular Chr III when there is no resection and, therefore, no PFGE shift in either 1st or 2nd dimensions. The gels were stained with SYBR Gold.
(TIF)

Figure S3 Resection of the “left side” of the *in vivo* I-*Sce* I cut chromosome III. Similar to Figure 2E, there is a bias for m* molecules that are not resected at the “left” side of the DSB. That is, there is a bias in the *Δrad50* mutant for resection of the “right” end when MRX is absent as compared to the opposite resection bias in the *Δsae2* and *mre11-nd* mutants when the complex is present.
(TIF)

Figure S4 1-end resection of I-*Sce*I-induced breaks in *Δrad50* *Δku70* cells. 2D-PFGE analysis of *Δrad50* and *Δrad50* *Δku70* using “right” fragment probe. The lower spot under the m* position confirms that even in the absence of Ku complex, structural MRX is required for coincident resection at an I-*Sce*I-induced DSB. Note: *Δrad50* is also included in Figure 2D and is shown here for comparison.
(TIF)

Table S1 Primers used for preparation of probes used in Southerns. Primer pairs were used for PCR amplification of genomic DNA. The appropriate PCR products were labeled with ^32^P-dCTP by random priming as described in [48]. The Cha1 primers were used for labeling of the circular chromosome 3 for IR-induced DSBs. Primers in the III45.5 to III50 series were used for preparation of probes specific for the left fragment of the I-*Sce*I+SfiI cut circular chromosome 3. All other primer pairs are specific for the right fragment.
(DOCX)
