# Supplementary figures and images for: Coincident Resection at Both Ends of Random, γ–Induced Double-Strand Breaks Requires MRX (MRN), Sae2 (Ctp1), and Mre11-Nuclease
Source: PLoS Genet. 2013 Mar 28;9(3):e1003420. doi: 10.1371/journal.pgen.1003420 (PMC3610664; doi:10.1371/journal.pgen.1003420)

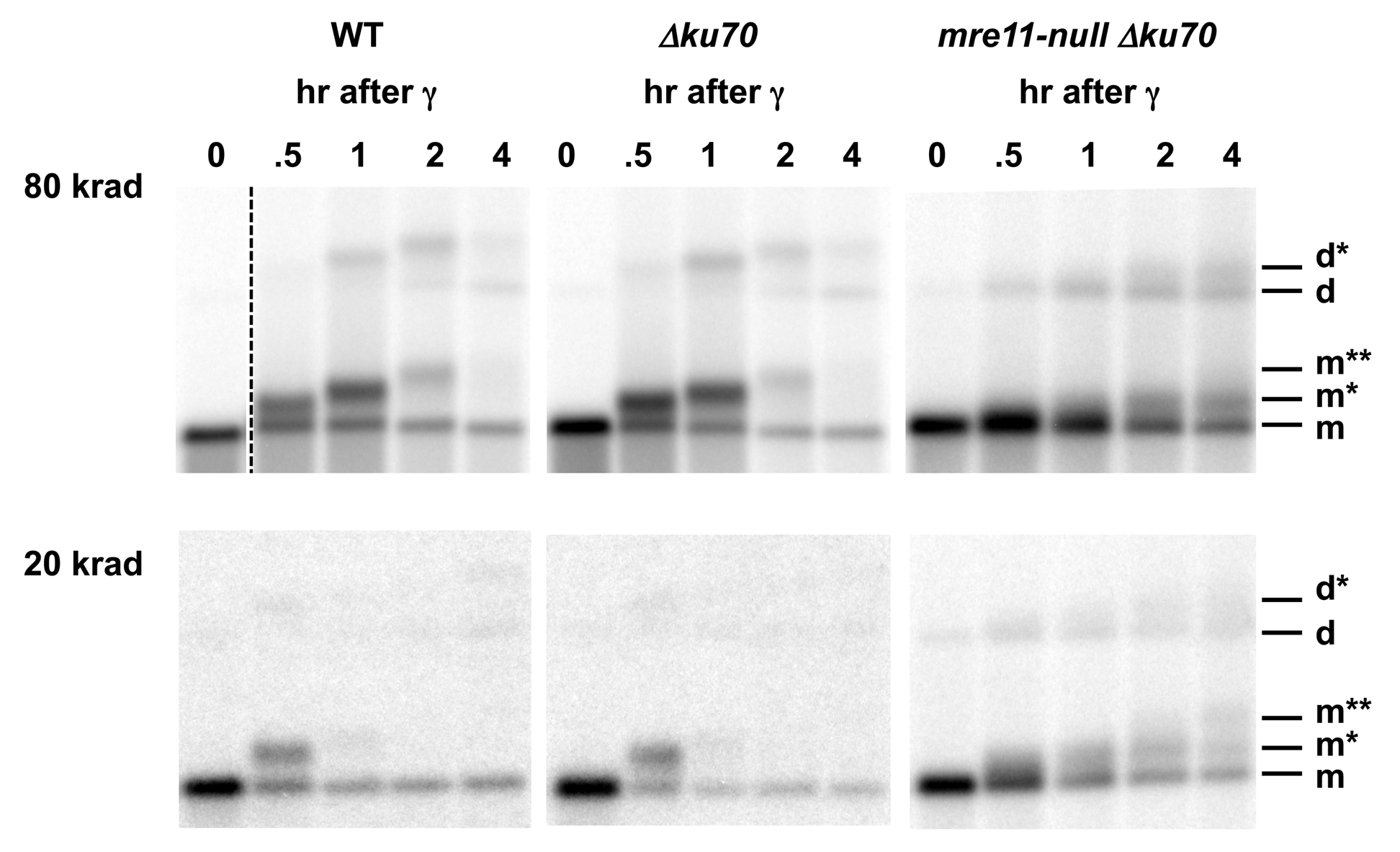

Supplement: Figure S1 — 1-end resection of radiation-induced breaks in mre11-null Δku70 cells. Presented are Southern blots of Chr III from cells arrested in G2 that were exposed to 80 krad and 20 krad. The PFGE-shift pattern for the mre11-null Δku70 double mutant strongly resembles that of the mre11-null and Δrad50 single mutants shown in Figure 1B and 1C. At both 80 and 20 krads, the presence m* molecules indicates 1-end resections, and fully shifted m** molecules are not detected after 80 krads. The WT and Δku70 strains have PFGE shift kinetics similar to Δrad51 (Figure 1A and 1C), indicating coincident resection at both sides of DSBs. Unlike Δrad51, these strains are repair proficient, so that the resected molecules diminish with time as they are recircularized by HDRR. After 20 krads, ∼80% of DSBs were repaired in the first hour (data not shown), so that the shifted band is very faint after 1 hour. (TIF) [file pgen.1003420.s001.tif]

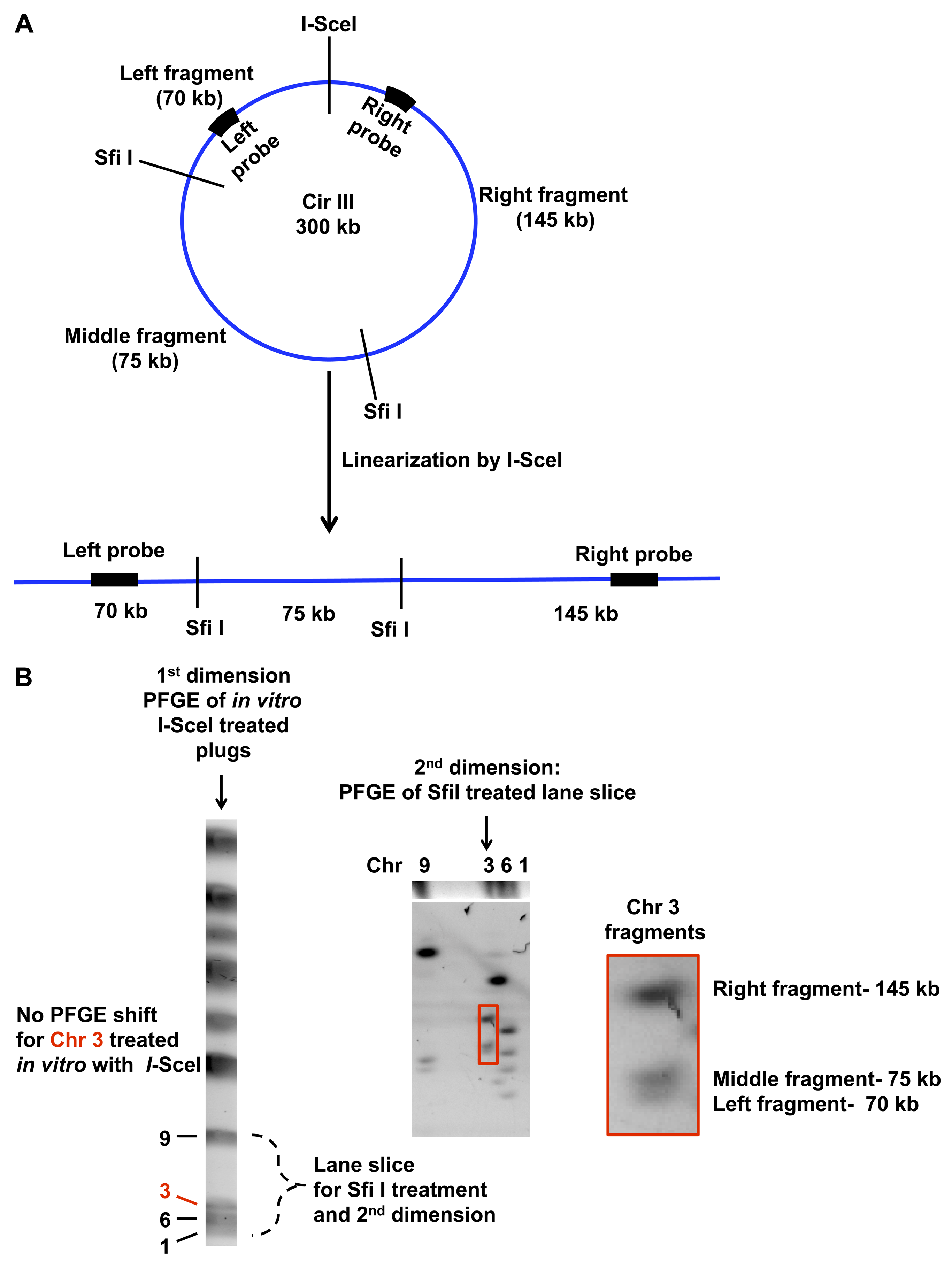

Supplement: Figure S2 — 2D-PFGE analysis of I-SceI cut site and SfiI restriction site targets in Chr III. A. Map of circular Chr III with I-SceI cut site and restriction site targets of the rare-cutter SfiI. B. 2D-PFGE pattern after in vitro I-SceI digest of plugs of unirradiated cells. An in vitro I-SceI digest of the Δrad50 strain containing an I-SceI site shows the positions of the three I-SceI/SfiI fragments of circular Chr III when there is no resection and, therefore, no PFGE shift in either 1st or 2nd dimensions. The gels were stained with SYBR Gold. (TIF) [file pgen.1003420.s002.tif]

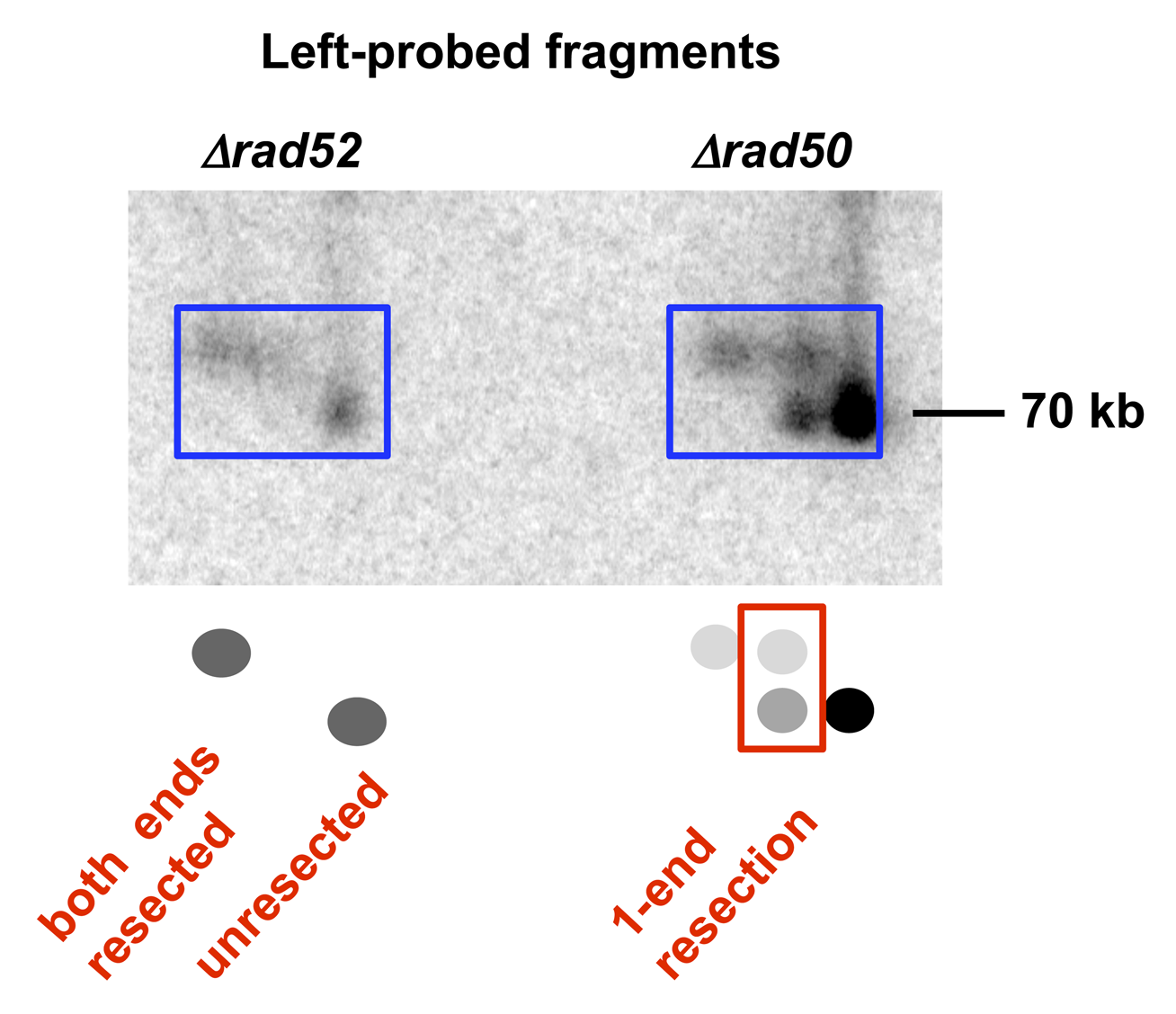

Supplement: Figure S3 — Resection of the “left side” of the in vivo I-Sce I cut chromosome III. Similar to Figure 2E, there is a bias for m* molecules that are not resected at the “left” side of the DSB. That is, there is a bias in the Δrad50 mutant for resection of the “right” end when MRX is absent as compared to the opposite resection bias in the Δsae2 and mre11-nd mutants when the complex is present. (TIF) [file pgen.1003420.s003.tif]

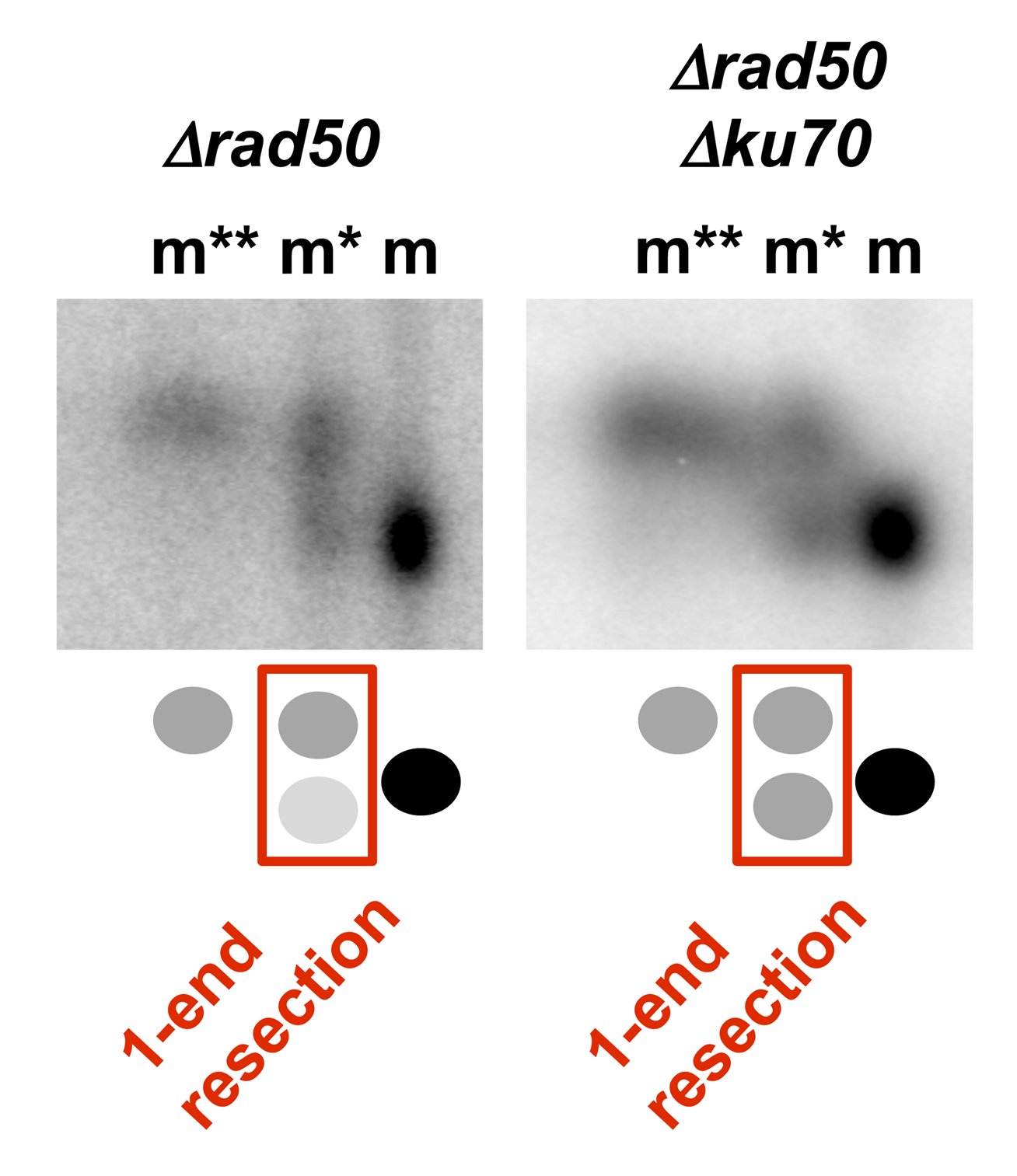

Supplement: Figure S4 — 1-end resection of I-SceI-induced breaks in Δrad50 Δku70 cells. 2D-PFGE analysis of Δrad50 and Δrad50 Δku70 using “right” fragment probe. The lower spot under the m* position confirms that even in the absence of Ku complex, structural MRX is required for coincident resection at an I-SceI-induced DSB. Note: Δrad50 is also included in Figure 2D and is shown here for comparison. (TIF) [file pgen.1003420.s004.tif]
