# Supplementary material for: Coincident Resection at Both Ends of Random, γ–Induced Double-Strand Breaks Requires MRX (MRN), Sae2 (Ctp1), and Mre11-Nuclease
Source: PLoS Genet. 2013 Mar 28;9(3):e1003420. doi: 10.1371/journal.pgen.1003420 (PMC3610664; doi:10.1371/journal.pgen.1003420)
Supplement: Table S1 — Primers used for preparation of probes used in Southerns. Primer pairs were used for PCR amplification of genomic DNA. The appropriate PCR products were labeled with 32P-dCTP by random priming as described in [48]. The Cha1 primers were used for labeling of the circular chromosome 3 for IR-induced DSBs. Primers in the III45.5 to III50 series were used for preparation of probes specific for the left fragment of the I-SceI+SfiI cut circular chromosome 3. All other primer pairs are specific for the right fragment. (DOCX) [file pgen.1003420.s005.docx]

**Table S1. Primers used for preparation of probes used in Southerns.**

| **Primer** | **Sequence** |
| --- | --- |
| Cha1-5’ | 5’- AACGGCCGTGATCTCTAATC-3’ |
| Cha1-3’ | 5’- TCCAACGCTTCTTCCAAGTC-3’ |
| Pgs1-5’ | 5’- GCTCCAACTCACTCGTCCTC-3’ |
| Pgs1-3’ | 5’- TTTCAACTTGGGGTTCTTGG-3’ |
| III50-fwd | 5’- TTGAAGTCGGCGTCAATCTT-3’ |
| III50-rev | 5’-TTGTTGCAAGCAGCATGTCT-3’ |
| III45.5-fwd | 5’-TCTAATTCCATAACGATTGCC-3’ |
| III45.5-rev | 5’- ACACTAGGTGACAAATCGGTT-3’ |
| III47.0-fwd | 5’- TAGCCGTCTGAAAAATCACG-3’ |
| III47.0-rev | 5’- TCAACGAGTACCTCAATCGCA-3’ |
| III47.6-fwd | 5’- TTGTATTCCAAGGGTCCGAA-3’ |
| III47.6-rev | 5’- ACTCGGATTTATCCTTTGGC-3’ |
| III48.2-fwd | 5’- TTATCAGGAGCGGGTACGA-3’ |
| III48.2-rev | 5’- TGAATTGGCTGACGAAGAAGA-3’ |
| III48.7-fwd | 5’- TTCCTCAGCAGCTTTTTCCT-3’ |
| III48.7-rev | 5’- ATCGTGTTGGAGTCTAAGGCT-3’ |
| III131-fwd | 5’- TGTTGAATCTGCGCTAAACGA-3’ |
| III131-rev | 5’- CATTGCCGAGGTCATCTCTAA-3’ |
| III133-fwd | 5’- GAAAATGCAGTCTTCCCCAA-3’ |
| III133-rev | 5’- TATTGAAGTTCCAGGCGCTA-3’ |
| III134-fwd | 5’- AAAGCACCAGCACGCTTAAT-3’ |
| III134-rev | 5’- CAATCTGCTGGTTTTCTGCAA-3’ |
| III135-fwd | 5’- CCACGATCAAATTCATTACCA-3’ |
| III135-rev | 5’- CAGATGAAGATGCGGTGAAT-3’ |
| III136-fwd | 5’- GTGGTATTTTGCCGTTCAAA-3’ |
| III136-rev | 5’- TCCTGCTCAGTGGTACTTGCA-3’ |
| III137-fwd | 5’- ACGGCTCACAGGTTTTGTAA-3’ |
| III137-rev | 5’- CAATGGGACGTTGAAGTCAA-3’ |
